# The WUR0000125 PRRS resilience SNP had no apparent effect on pigs’ infectivity and susceptibility in a novel transmission trial

**DOI:** 10.1186/s12711-023-00824-z

**Published:** 2023-07-24

**Authors:** Margo Chase-Topping, Graham Plastow, Jack Dekkers, Yanhua Li, Ying Fang, Volker Gerdts, Jill Van Kessel, John Harding, Tanja Opriessnig, Andrea Doeschl-Wilson

**Affiliations:** 1 The Roslin Institute, University of Edinburgh, Easter Bush, Roslin, Edinburgh, UK; 2 Department of Agricultural, Food and Nutritional Science, University of Alberta, Edmonton, AB Canada; 3 Department of Animal Science, Iowa State University, Ames, IA USA; 4 Department of Diagnostic Medicine/Pathobiology, Kansas State University, Manhattan, KS USA; 5 Department of Pathobiology, University of Illinois Urbana-Champaign, Champaign, IL USA; 6 Vaccine and Infectious Disease Organization-International Vaccine Centre, University of Saskatchewan, Saskatoon, Canada; 7 Department of Large Animal Clinical Sciences, University of Saskatchewan, Saskatoon, Canada; 8 Vaccines and Diagnostics Department, Moredun Research Institute, Penicuik, UK; 9 Department of Veterinary Diagnostic and Production Animal Medicine, Iowa State University, Ames, IA USA

## Abstract

**Background:**

Porcine reproductive and respiratory syndrome (PRRS) remains one of the most important infectious diseases for the pig industry. A novel small-scale transmission experiment was designed to assess whether the WUR0000125 (WUR for Wageningen University and Research) PRRS resilience single nucleotide polymorphism (SNP) confers lower susceptibility and infectivity to pigs under natural porcine reproductive and respiratory syndrome virus (PRRSV-2) transmission.

**Methods:**

Commercial full- and half-sib piglets (n = 164) were assigned as either Inoculation, Shedder, or Contact pigs. Pigs were grouped according to their relatedness structure and *WUR* genotype, with R− and R+ referring to pigs with zero and one copy of the dominant *WUR* resilience allele, respectively. Barcoding of the PRRSV-2 strain (SD09-200) was applied to track pig genotype-specific transmission. Blood and nasal swab samples were collected and concentrations of PRRSV-2 were determined by quantitative (q)-PCR and cell culture and expressed in units of median tissue culture infectious dose (TCID_50_). The Log_10_TCID_50_ at each sampling event, derived infection status, and area under the curve (AUC) were response variables in linear and generalized linear mixed models to infer *WUR* genotype differences in Contact pig susceptibility and Shedder pig infectivity.

**Results:**

All Shedder and Contact pigs, except one, became infected through natural transmission. There was no significant (p > 0.05) effect of Contact pig genotype on any virus measures that would indicate *WUR* genotype differences in susceptibility. Contact pigs tended to have higher serum AUC (p = 0.017) and log_10_TCID_50_ (p = 0.034) when infected by an R+ shedder, potentially due to more infectious R+ shedders at the early stages of the transmission trial. However, no significant Shedder genotype effect was found in serum (p = 0.274) or nasal secretion (p = 0.951) that would indicate genotype differences in infectivity.

**Conclusions:**

The novel design demonstrated that it is possible to estimate genotype effects on Shedder pig infectivity and Contact pig susceptibility that are not confounded by family effects. The study, however, provided no supportive evidence that genetic selection on *WUR* genotype would affect PRRSV-2 transmission. The results of this study need to be independently validated in a larger trial using different PRRSV strains before dismissing the effects of the *WUR* marker or the previously detected *GBP5* gene on PRRSV transmission.

**Supplementary Information:**

The online version contains supplementary material available at 10.1186/s12711-023-00824-z.

## Background

Infectious diseases can drastically reduce production performance, fertility, and survival in farm animals. Therefore, they constitute a huge threat to the sustainability and profitability of livestock production and to carbon neutral farming [1]. They also increase the threat of antimicrobial resistance, as it is often necessary to treat animals with antimicrobials to overcome disease and reduce mortality [2]. Porcine reproductive and respiratory syndrome (PRRS) arguably remains one of the most important infectious diseases for the pig industry worldwide. The etiological agent is a positive-sense RNA virus with two major species (formally genotypes): porcine reproductive and respiratory syndrome virus (PRRSV) PRRSV-1 (*Betaarterivirus suid 1*, formerly known as the European PRRSV-1) and PRRSV-2 (*Betaarterivirus suid 2*, formerly known as the North American PRRSV-2) [3, 4]. The disease is commonly associated with reproductive failure, respiratory disease, fever, reduced growth, and mortality in piglets and breeding animals. It is also known to enhance the risk of secondary infections by other pathogens. In spite of biosecurity measures and vaccination, PRRS prevalence continues to be high, with estimated production losses of around 860 and 1660 million euros per year for the US and Europe, respectively [5]. The difficulty in controlling PRRS is largely driven by the high mutation rate of the virus, giving rise to a high diversity of virus strains which jeopardizes the effectiveness of preventative vaccines and medications [6].

One of the proposed strategies to mitigate production losses associated with PRRS is to breed animals that are genetically more disease resilient, i.e. that maintain high performance levels in the face of pathogen challenge [7–11]. Improved disease resilience can be achieved in multiple ways, as disease resilience captures complementary host defence mechanisms against pathogens, such as host resistance and tolerance [5, 12]. Disease resistance is the ability of a host animal to limit within-host pathogen load, whereas tolerance is the ability of an infected animal to limit the reduction in performance or fitness associated with a given within-host pathogen load [13]. There is increasing awareness that an effective selection program for disease resilience must also reduce pathogen transmission within and between farms, as herd resilience also depends on the pathogen load in the environment [14]. Pathogen transmission is largely controlled by two host traits: susceptibility to becoming infected, and infectivity, the capacity of an animal, once infected, to transmit infection to others. Less susceptible animals are less likely to become infected in the first place and thus are also less likely to transmit infection. Infectivity is likely related to pathogen shedding and, thus, selecting animals with lower infectivity is expected to reduce the pathogen challenge level in the herd, resulting in fewer infections and associated production losses.

Many studies have shown that there is substantial genetic variation in the resistance, tolerance, and resilience of pigs to PRRSV infection, and that these traits are polygenic [8]. While it seems reasonable to expect that pigs also vary genetically in their PRRSV susceptibility and infectivity, to date, estimates of the genetic variances and the genomic architecture for these traits are not available, as the methods required for the genetic study of these traits are still in their infancy [14].

Although natural genetic variation in resilience traits offers prospects for PRRS control through genetic improvement, direct selection for resilience traits is difficult because the elite nucleus populations on which genetic selection is practiced must be kept under biosecure high-health conditions, and thus animals are not exposed to high levels of infectious pathogens [8]. An alternative approach is to select on genes or genetic markers linked to genes that are predictive of disease resilience, as this can be practiced in high-health nucleus herds. A series of large-scale PRRSV challenge studies conducted by the PRRS Host Genetic Consortium (PHGC) [15] led to the discovery of the single nucleotide polymorphism (SNP) WUR0000125 (*WUR*) on porcine (*Sus scrofa*) chromosome (SSC) 4 that is associated with resilience, resistance, and tolerance of pigs inoculated with PRRSV. This SNP accounted for 11.2% of the genetic variance for host resilience as measured by weight gain to 42 days post-infection (dpi) and for 15.7% of genetic variance in resistance as measured by serum viral load from 0 to 21 dpi [16, 17]. Compared to individuals that carry the unfavourable homozygous genotype at the *WUR*-SNP, nursery pigs that were heterozygous grew on average 2 kg more in the 42 dpi, had on average a 4.5% lower viral load within 21 dpi, and grew on average 10 g/day more per unit increase in viral load [16–18]. The favourable allele also appeared to be dominant, i.e. one copy of the favourable allele was sufficient to achieve the above effects [17].The underlying molecular mechanisms regulated by the quantitative trait locus (QTL) are yet poorly defined [19, 20]

Although the PHGC challenge experiments led to the identification of the *WUR* PRRS resilience SNP, its role in establishing herd resilience for pigs that are naturally exposed to PRRSV still needs to be fully established. A recent natural polymicrobial disease challenge study, in which more than 3000 piglets were placed in a grow-finish facility that was seeded with multiple pathogens, including PRRSV-2, showed that pigs carrying at least one favourable *WUR* resilience allele had a higher growth rate, required fewer health treatments, and had a lower tendency to die [11]. This study suggests that the favourable *WUR* SNP allele also confers greater resilience to pigs that are naturally exposed to PRRSV-2 along with other pathogens. However, to date, the role of the *WUR* SNP on natural PRRSV transmission is not elucidated. In particular, it is not known whether the *WUR* genotype confers differences in pig susceptibility and infectivity in horizontally-exposed populations, which is typical of endemic field conditions.

The overall aim of this study was to determine the effect of the *WUR* resilience SNP on PRRSV-2 transmission. Towards this purpose, a novel transmission experiment was designed that differed from conventional transmission experiments in two key aspects: (i) use of a specific genetic relatedness structure and *WUR* genotype composition of pigs in the different contact groups to maximise statistical power to estimate genotype effects on PRRSV transmission, and (ii) barcoding of the PRRS-2 virus to trace specific transmission by pigs with different *WUR* genotypes.

## Methods

### Transmission experiment

**Fig. 1:**
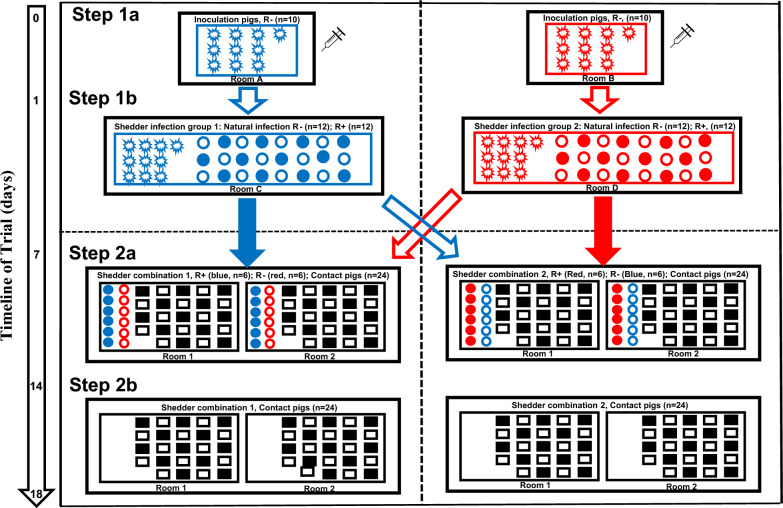
Experimental design. Scheme of the design of the trial, comprising the creation of the naturally-infected Shedder pigs (Step 1) for use in the main transmission trial (Step 2). Step 1a. direct infection of the Inoculation pigs (day 0; challenge dose was 10^5^ TCID_50_ in a total volume of 4 mL, and the challenge route was intramuscularly (IM) and intra-nasally (IN) with 2 mL IM and 1 mL for each nostril) and Step 1b. infection of Shedder pigs through natural transmission (day 1 to day 6); and Step 2a. re-assortment of the naturally infected Shedder pigs and placement in rooms with naïve Contact pigs (day 7) and Step 2b. removal of the Shedder pigs (day 14). Jagged circles: directly Inoculation pigs; Smooth circles, naturally infected Shedder pigs; Squares, Contact pigs; closed symbol, R+ genotype; open symbol, R− genotype3; Blue, Shedder infection group 1; Red, Shedder infection group 2. Arrows indicate the direction of the movement of pigs (R+ solid arrows; R− open arrows)

The aim of the novel design was to provide unbiased estimates for genotype differences in Shedder pig infectivity and Contact pig susceptibility that are not confounded by potential family effects on these traits. The study design consisted of two steps (Fig. 1): step 1 involved the creation of Shedder pigs through natural exposure to Inoculation pigs that were directly infected with PRRSV-2, while step 2 was the main transmission trial. In total, 164 commercial cross-bred post-weaning barrows (full-sibs or paternal half-sib progeny from 51 Large White sows and 24 Landrace sires), up to 42 days old, were used in this study. Piglets, all PRRSV negative and from the same farm (Olymel—Engdahl), were sourced at approximately 3 weeks of age from Hypor Canada. The *WUR* genotypes of piglets were determined and the piglets were assigned to groups and rooms prior to movement to the study site (Vaccine and Infectious Disease Organization (VIDO)/International Vaccine Centre (InterVac), Saskatoon, Saskatchewan). On arrival, each piglet was given an ear-tag and one intramuscular dose of Excede 100 Sterile Suspension (5.0 mg/kg; Ceftiofur Crystalline Free Acid Zoetis^®^), as per label instructions. Animals were kept for one week before the start of the trial to acclimatize.

To avoid confounding between genotype and family effects, the trial used balanced sibling groups consisting of four full-sibs or paternal half-sibs. Approximately half of the pigs from each sibling group carried (genotype: R+) or did not carry (genotype: R−) a copy of the dominant *WUR* resilience allele. After considering parentage and genotype (Fig. 1 and as outlined below), pigs were randomly assigned to one of the following roles: (1) Inoculation pig (n = 20); (2) Shedder pig (n = 48); and (3) Contact pig (n = 96). Inoculation pigs (all R−, 9 full-sib pairs and 1 unrelated pair) were split between two rooms, where they were inoculated with either a bar-coded (room A, blue, Step 1a in Fig. 1) or a non-barcoded (room B, red, Step 1a in Fig. 1) version of a PRRSV-2 isolate (SD09-200; infection dose 10^5^ TCID_50_ in a total volume of 4 mL; intramuscularly (IM) and intra-nasally (IN) with 2 mL IM and 1 mL in each nostril). The SD09-200 virus was originally isolated from a serum sample that was obtained from a US swine farm in 2009, in which nursery pigs were experiencing respiratory disease. The barcoded virus was constructed by introducing synonymous mutations into the non-structural protein nsp1β or nsp2 region and the recombinant virus was obtained by using reverse genetics as described previously [21]. The barcode enables tracking of shedder genotype-specific transmission routes for estimating differences in infectivity between R+ and R− Shedder pigs. Barcoding has been used successfully in other experimental infection studies [22–24]. A pilot experiment (see Additional file 1: Text S1 and Tables S1, S2, S3 and S4) in which a small number of pigs (n = 16) were infected with either a barcoded or a non-barcoded version of the virus showed that the barcoding had no effect on the infectivity or pathogenesis of the virus (i.e. no difference in virus load in serum, nasal swabs, or lungs, and no difference in temperature or other clinical signs) (see Additional file 2: Text S2 and Figures S1, S2, S3, and S4). In addition, the virus remained genetically intact in the targeted barcode regions in the serum of challenged piglets (M. Liao and J. Van Kessel personal communication).

One day post-infection of the Inoculation pigs, 24 Shedder pigs from 12 sibling groups (n = 4 per group; 36 full-sibs; 12 paternal half-sibs) were introduced into two rooms (each consisting of equal proportions of R+ and R– pigs and with sibs of the same R genotype split across rooms), where they were in direct contact with the Inoculation pigs (Step 1b in Fig. 1). At day 7, naturally-infected Shedder pigs were transferred into four new rooms (Step 2a in Fig. 1) according to their R genotypes and sibling group (sibs of the same R genotype were split across rooms to avoid confounding between relatedness and R genotype effects), where they could transmit PRRSV-2 to resident naïve Contact pigs (24 sibling groups; n = 4 per group; 88 full-sibs; 8 paternal half-sibs). Prior to transfer, qPCR confirmed that the naturally infected Shedder pigs contained detectable levels of virus in their serum. In addition, qPCR analyses of the Inoculation pigs showed that there was no significant difference in serum viral load between the two Inoculation groups (see Additional file 3: Figure S5). To minimise re-infection of Shedder pigs, they were removed at day 14 (Step 2b in Fig. 1). The trial lasted only 18 days, which is sufficient to ensure that Contact pigs were infected by Shedders, and Contact-to-Contact pig transmission was unlikely.

### Sampling and virus determination

Table 1 contains the timeline of the study, including when samples were taken from Inoculation, Shedder, and Contact pigs. Sampling involved collecting nasal mucus samples using swabs and blood samples on trial day (d) 1, 6, 8, 9, 10, 14, and 17 (Table 1). Serum was isolated, and nasal swabs were stored in 0.5 mL Minimum Essential Medium (MEM) medium with antibiotics. Samples were collected from Inoculation pigs on day 1 to confirm the infection status (see Additional file 3: Figure S5). Sample time was standardised for Shedder and Contact pigs. Samples were collected from Shedder pigs on days post-contact (dpc) 5, 7, 9, and 13 with the directly infected Inoculation pigs (dpc_S) and samples were collected from Contact pigs on dpc 2, 7, and 10 with the naturally-infected Shedder pigs (dpc_C). The timing of samples captured expected early and mid stages of infection, including time of peak viremia [25, 26]. Piglets were weighed at the beginning and the end of the trials. Animals were observed daily for clinical presentations and clinical scores. Body temperature was measured daily for 7 days post-challenge. At necropsy, lymph nodes and thymus samples were collected. All samples were stored at −80 °C before shipping to the University of Alberta for further analysis.

**Table 1:** Timeline for the transmission trial

		Trial (day)	Shedder (dpc_S)	Contact (dpc_C)	Event
Step 1	A	0			Direct infection of Inoculation pigs
		*1*	0		Sample, transfer Inoculation pigs; introduce naïve Shedder pigs
		2	1		–
	B	3	2		–
		4	3		–
		5	4		–
		*6*	*5*		Sample Inoculation and Shedder pigs
Step 2	A	7	6	0	Transfer infected Shedder pigs; introduce naïve Contact pigs
		*8*	7	1	Sample Shedder pigs
		*9*	8	*2*	Sample Contact pigs
		*10*	*9*	3	Sample Shedder pigs
		11	10	4	–
		12	11	5	–
		13	12	6	–
	B	*14*	*13*	7	Remove Shedder pigs; sample Shedder & Contact pigs
		15	14	8	–
		16	15	9	–
		*17*	16	*10*	Sample Contact pigs
		18	17	11	Termination of trial

Timing of events carried out during the 18 day trial, standardised for Shedder pigs (S) and Contact pigs (C) for the time (days) since exposure to infected donors (days post contact (dpc)). Step 1, Infection of the Shedder pigs Step 1A, direct infection of the Inoculation pigs; Step 1B, infection of Shedder pigs through natural transmission; Step 2, Main transmission trial (Step 2A, naturally infected Shedder pigs introduced to naive Contact pigs; Step 2B, Shedder pigs removed); dpc_S, for Shedder pigs, days post contact from directly infected Inoculation pigs; dpc_C, for Contact pigs, days post contact from naturally infected Shedder pigs. Sample refers to serum and nasal swab samples taken. Sample dates are highlighted in italics. -, no event

PRRS viral titres in serum and nasal secretions were determined using both cell culture and qPCR. Full details on the qPCR analysis are in Additional file 1: Text S1. MARC145 cells were used for the cell culture method. Cells were re-suspended in MEM medium containing the appropriate concentration of foetal bovine serum (FBS) to a concentration of 1 × 10^6^ to 1.5 × 10^7^ cells/mL. Cells were cultured in 96-well plates to confluence. Samples were serially diluted 1:10 in MEM media and were added to each well, after which cells were incubated for 1 h at 37 °C for the establishment of infections. Cells were incubated with fresh MEM medium at 37 °C for 2–5 days and observed for cytopathic effect (CPE). Each assay was performed in triplicate. Values of TCID_50_ were calculated using the Reed and Muench method [27].

Viruses from the virus stocks and serum samples were sequenced for the expected bar-code regions. cDNA was prepared from the virus RNA templates at VIDO-InterVac and amplified by PCR using the sequencing primer pair (see Additional file 1: Text S1 for full details), and then sent to the Plant Biotechnology Institute of The National Research Council (NRC-PBI). Details on the primers used for PCR amplification and cDNA sequencing are in Additional file 1: Text S1 and Tables S2, S3 and S4.

### Statistical analyses

Statistical analyses were performed on data from all 96 Contact pigs and 48 Shedder pigs used in step 2 of the trial, unless stated otherwise. Response variables for the statistical models included the area under the curve (AUC) of the log_10_TCID_50_ viral load (generated using the spline rule (R version 4.1.0)), the log_10_TCID_50_ values at each sampling event, and the proportion of pigs with positive viral load. To avoid confounding between room in Step 1 and the two versions of the PRRSV, the variable ‘shedder infection group’ (2-level category; barcoded, blue vs non-barcoded, red in Fig. 1) was created. Similarly, the variable ‘shedder combination’ (2-level category; separated by the dotted vertical line in Fig. 1) was created to represent the different combinations of shedder R genotype and shedder infection group that the Contact pigs were exposed to.

Agreement between virus detection, composition, and quantity between nasal and serum samples was assessed in Shedder pigs. Various statistical models were applied to assess the effect of the pig’s R genotype on Shedder pig infectivity and Contact pig susceptibility. Genotype effects on Shedder pigs’ infectivity can manifest as a difference at a given time in the proportion of infected Shedder pigs that shed the virus (as indicated by positive viral load from nasal swabs), or in a difference in the number of Contact pigs that are infected by R+ versus R− Shedder pigs (as determined by the bar-code of the virus isolated from the serum of the Contact pigs). Genotype effects on Contact pig susceptibility can manifest as a difference in the proportion of infected R+ versus R− Contact pigs, or in the difference in the time of infection of R+ versus R− Contact pigs (i.e. more susceptible pigs tend to become infected earlier). Furthermore, serum and nasal viral load can be important indicators for Shedder pig infectivity, as more infectious pigs are expected to harbour and shed more virus. Similarly differences in serum viral load can be indicative of differences in Contact pig susceptibility, as the virus may be able to replicate faster in more susceptible pigs.

In line with these concepts, using linear mixed models (LMM, SAS Proc Mixed), analysis of R genotype effects on viral load was performed on Shedder (S) and Contact (C) pig serum AUC (AUC_S_ and AUC_C_, respectively) and log_10_TCID_50_ values, and on nasal AUC_S_ and log_10_TCID_50_ values of Shedder pigs at the different sampling events. Genotype (Shedder and Contact pig models), shedder infection group (Shedder pig models), shedder combination (Contact pig models), and day (log_10_TCID_50_ models) were included as fixed effects, along with the initial weight of pigs as a covariate. In addition, to account for potential differences in the level of exposure in each room (in step 2, Fig. 1), total (sum) Shedder nasal log_10_TCID_50_ in each room during the observation period was included as a covariate in all Contact pig models. Room differences in exposure can also be accounted for by including room nested within shedder combination as a fixed effect in the statistical model but this resulted in a poorer model fit (higher Bayesian Information Criterion (BIC)) than including level of exposure as a covariate. All relevant interactions were tested but removed if they did not improve the overall fit of the model (that was assessed based on improvement in the BIC). An unstructured covariance pattern was added to the residuals in the Shedder and Contact temporal models for log_10_TCID_50_ to account for correlations between the repeated measurements. Other covariance patterns were tested (compound symmetry, unstructured, autoregressive AR(1), toeplitz and spatial power) but the unstructured pattern provided the best model fit (based on BIC).

Models used to assess the effects of R genotype on Shedder pig infectivity also included temporal analyses of the probability of nasal shedding, as determined by a positive nasal viral load, and of the probability of early (2 dpc_C) infection of Contact pigs, as well as of the overall probability of infection by a R+ vs R− Shedder pig at the end of the trial. These models were performed using a generalised linear mixed model (GLMM) (SAS, Proc Glimmix), with the same fixed effects as described above, and presence or absence of detectable virus in serum or nasal mucus as the corresponding response variables. Similar to the LMM above, differences in exposure were accounted for by including the total (sum) nasal log_10_TCID_50_ of all infected shedder pigs in the same room as covariates. For 2 dpc_C, total shedder nasal log_10_TCID_50_ from 7 dpc_S was used, while total R+ shedder nasal log_10_TCID_50_ for the entire sampling period was added for 10 dpc_C.

Unless stated otherwise, all statistical analyses were conducted using SAS version 9.4 (SAS Institute, Cary, NC). Sibling-group was added as a random effect in all models to account for genetic effects on the response variables that were not captured by R genotype. Statistical significance was set at p < 0.05 and BIC was used as a measure of model fit.

## Results

### Variation between and within sibling groups

**Fig. 2:**
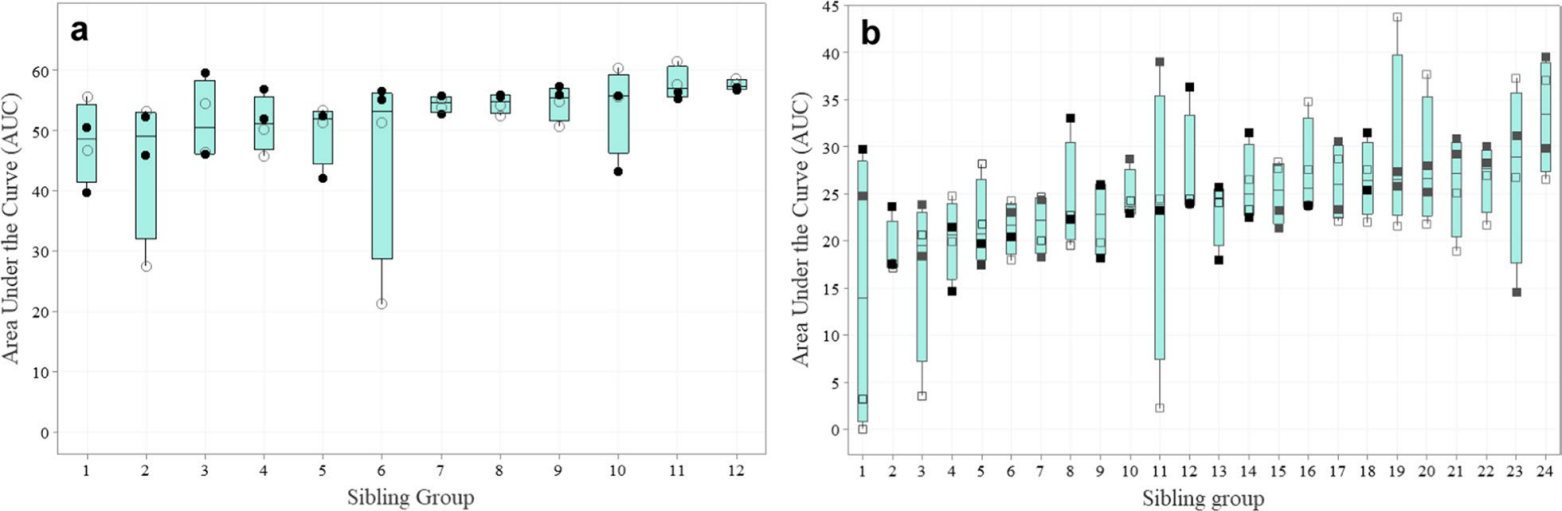
Differences in serum viral load as measured by area under the curve (AUC) for **a** shedder pigs (n = 12) and **b** contact pigs (n = 24) sibling groups. Data are organized based on increasing median AUC. Open circles/squares, R− genotype; closed circles/squares, R+ genotype

Adding a random effect of sibling group improved the fit (lower BIC) of all models, suggesting the presence of genetic and/or common environmental variation in the response variables. Nevertheless, variation across sibling groups was generally low. For example, for serum AUC, sibling groups accounted for only 12% (AUC_S_) and 14% (AUC_C_) of the variance, and for only 7% of the variance of nasal AUC_S_ (see Additional file 4: Text S3 and Tables S5 and S6). The remaining variation (more than 80%) was attributable to individuals within the Shedder and Contact pig sibling groups. There appeared to be no systematic effect of the R genotypes based on serum AUC (Fig. 2a and b). As shown in the figure, R+ Shedder and Contact pigs did not have systematically lower AUC values than R− pigs, as expected based on previous literature [8, 16].

### General infection and shedding patterns

#### Shedder pigs

**Fig. 3:**
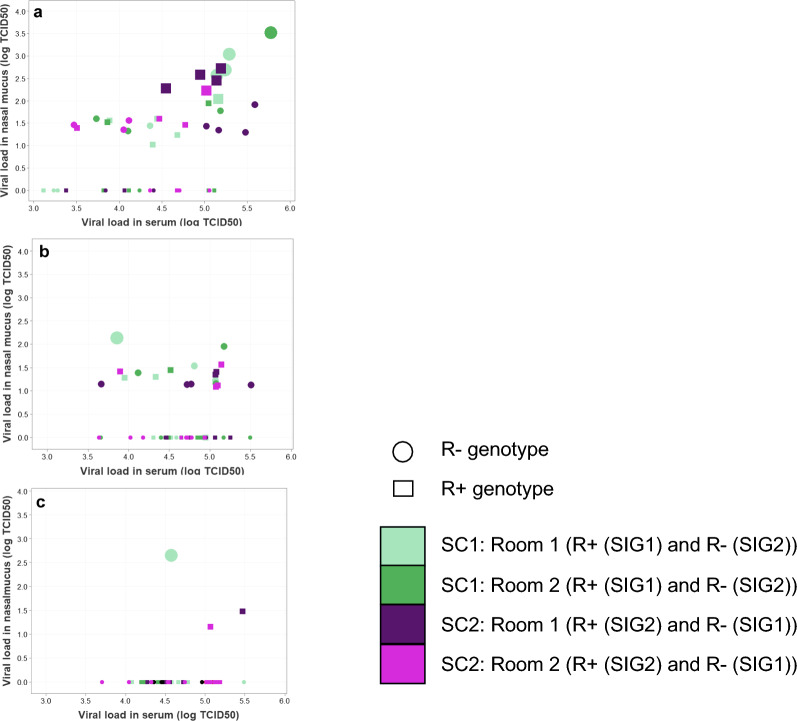
Relationship between viral load in the serum and viral load in the nasal mucus (log_10_TCID_50_) for all Shedder pigs (n = 177 pigs) at 7 dpc_S (**a**), 9 dpc_S (**b**) and 13 dpc_S (**c**). SC: Shedder combination; SIG: Shedder infection group; R+: one copy of the *WUR* resilience allele; R−: no copy of the *WUR* resilience allele; light green, SC1, replicate 1; dark green, SC1, replicate 2; dark purple, SC22, replicate 1; light purple, SC2, replicate 2); small symbol: zero log_10_TCID_50_; medium symbol: greater than 1 and less than 2 log_10_TCID_50_; large symbol: > 2 log_10_TCID_50_); closed circle: R+, one copy of the *WUR* resilience allele; open circle: R−, no copy of the *WUR* resilience allele. SIG1 were exposed to the barcoded PRRSV-2 and SIG2 were exposed to the identical PRRSV-2 without the barcode

All Shedder pigs were viremic (i.e. virus was detected in their serum) at 7, 9, and 13 dpc_S, while only two pigs were not viremic at 5 dpc_S (see Additional file 5: Figure S6). The log_10_TCID_50_ value of viremic pigs was consistently above 2 (see Additional file 6: Figure S7). At all time-points, the virus detected in all Shedder pigs’ serum was consistent with the virus that they were originally exposed to in their shedder infection group (see Additional file 5: Figure S6). Although all Shedders were viremic at 7 dpc_S, only 62.5% (n = 30/48) appeared to be shedding the virus (i.e. detectable virus in nasal mucus) from the upper respiratory tract (see Additional file 6: Figure S8) and (Fig. 3). This proportion decreased during the study, with 39.5% (n = 19/48) shedding at 9 dpc_S but only 6.35% (n = 3/48) at 13 dpc_S (see Additional file 6: Figure S8); and (Fig. 3). Comparison of nasal and serum virus levels for Shedder pigs with positive nasal swab and serum samples showed that virus levels were consistently lower in the nasal mucus than in the serum (Fig. 3). Furthermore, on 7 dpc_S, i.e. at the onset of the transmission study (step 2, Fig. 3), there was considerable variation in the level of virus detected in the nasal mucus (see Additional file 6: Figure S8), with high levels of shedding from several R+ Shedders in Shedder combination 2 and R− Shedders in Shedder combination 1, both originating from Shedder infection group 2, (Fig. 3) and (see Additional file 6: Figures S7 and S8).

#### Contact pigs

**Fig. 4:**
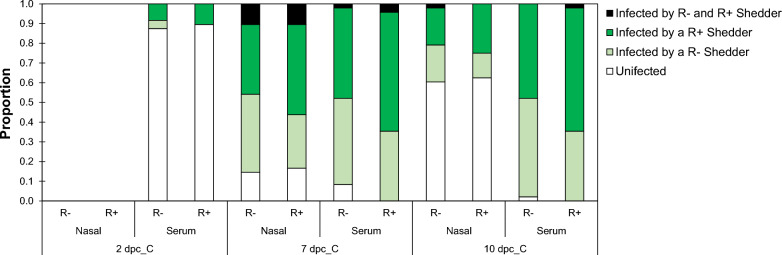
Proportion of contact pigs of each genotype. R+ : one copy of the *WUR* resilience allele; R−: no copy of the *WUR* resilience allele; dpc_C: Contact pigs, days post contact from an infected Shedder pig; white: uninfected; light green: infected a R− Shedder pig; green, infected by a R+ Shedder pig; dark green: infected by both R+ and R− Shedder pigs

Of the 96 Contact pigs used in the study, 95 (99%) were viremic by the end of the study (trial day 18, Contact pig 10dpc_C) and 92 (96%) were viremic by trial day 14 (Contact pig 7 dpc_C) (Fig. 4) and (see Additional file 7: Figure S9). On trial day 9 (Contact pig 2dpc_C), only 11 (11.5%) Contact pigs had detectable levels of virus in the serum (Fig. 4) and (see Additional file 7: Figure S9). Across all time points, only three Contact pigs had viruses from both R Shedder genotypes in their serum (Fig. 4). These pigs had similar log_10_TCID_50_ values for both viruses, so their log_10_TCID_50_ were averaged for the sampling date. Once infected by a given R Shedder, there was no evidence (based on serum samples) that Contact pigs were subsequently infected by a virus from the other R Shedder genotype (see Additional file 7: Figure S9).

As for the Shedder pigs, virus levels were generally lower and less stable in the nasal mucus than in the serum samples. In particular, no virus was detected in the nasal mucus of 100% (n = 11/11), 14% (n = 13/95), and 62% (n = 59/95) of the infected Contact pigs at 2 dpc_C, 7 dpc_C, and 10 dpc_C, respectively (see Additional file 8: Figure S10). Agreement between the R genotype observed in the serum and in the nasal mucus of Contact pigs was generally high (see Additional file 8: Figure S10). However, among the Contact pigs that were positive for both nasal swabs and serum at any one time point (n = 117), there was a small number (n = 4/117, 3.4%) for which the virus from the other Shedder genotype than the one they were infected with was detected in the nasal swabs (see Additional file 8: Figure S10). In addition, viruses from both genotypes were detected in the nasal swab samples of eight Contact pigs (n = 8/117, 6.7%), although virus from only one Shedder genotype was detected in the serum samples of these pigs (Fig. 4) and (see Additional file 8: Figure S10). These Contact pigs likely acquired the virus through nasal contact with various infected Shedder pigs, but not all of these resulted in infection.

### Indicators of infectivity for Shedder pig

Table 2 contains the results of the LMM for the serum AUC_S_ and nasal AUC_S_ of Shedder pigs. There was no significant genotype effect (serum: p = 0.27; nasal: p = 0.95) on Shedder viral load, regardless of the Shedder infection group from which the pigs originated. Serum viral load tended to be higher in Shedder Infection group 1 (p = 0.07) and in Shedder pigs with a smaller initial body weight (p = 0.04).

**Table 2:** Estimates from the linear mixed model of the effects on area under the curve of Shedder pigs (AUC_S_) for serum and nasal swab samples

Effect	Estimate	se	p-value
Serum AUC_s_			
Initial pig weight (log_10_ kg)	− 21.9	10.21	0.037
Shedder infection group (1 vs 2)	3.52	1.88	0.070
Shedder genotype (R− vs R+)	− 2.11	1.90	0.274
Nasal swab AUC_s_			
Initial pig weight (log_10_ kg)	− 7.99	7.84	0.313
Shedder infection group (1 vs 2)	− 0.624	1.48	0.675
Shedder genotype (R− vs R+)	− 0.092	1.49	0.951

Estimate: Parameter estimate; se: standard error; t: t-value and 2 tailed p-value used in testing the null hypothesis that the coefficient/parameter is 0; R− and R+ : pigs with zero and one copy of the *WUR* resilience allele, respectively

Table 3 shows the result of the LMM for Shedder serum and nasal log_10_TCID_50_ by time. There was no significant effect of Shedder genotype on log_10_TCID_50_ neither for nasal swabs (p = 0.98) nor for serum (p = 0.44). However, the Shedder infection group had a significant effect on both serum and nasal virus load. Nasal viral load of Shedders from group 2 had, on average, higher log_10_TCID_50_ values than group 1 (p = 0.004). The serum viral load from a Shedder infection group depended on the day of sampling (p = 0.002). At 5 dpc_S, the log_10_TCID_50_ of Shedder pigs in infection group 2 was significantly lower than that of Shedder pigs in infection group 1 (p = 0.008), because several Shedder pigs from group 2 had no or low levels of detectable virus in their serum at that stage (see Additional file 5: Figure S6). However, on 13 dpc_S, Shedder pigs from infection group 2 had, on average, significantly higher serum log_10_TCID_50_ (p = 0.002). There was also a significant day effect on nasal viral load, which was significantly higher at 7dpc_S (p < 0.001) than at 9 dpc_S and 13 dpc_S (Table 3). At later days, nasal viral load dropped below detection levels, especially for most Shedder pigs from infection group 1 (see Additional file 5: Figure S6).

**Table 3:** Linear mixed model (LMM) for Shedder pig serum PRRSV-2 log_10_TCID_50_ at different sampling days for serum and nasal swab samples

Effect	Estimate	se	t	p-value
Serum				
Initial pig weight (log_10_ kg)	− 0.359	0.432	− 0.83	0.407
Shedder infection group (1 vs 2)	0.021	0.194	0.110	0.912
Shedder genotype (R− vs R+)	− 0.069	0.090	− 0.770	0.442
Day (relative to 7 dpc_S)				
5 dpc_S	− 0.121	0.237	− 0.51	0.611
9 dpc_S	0.285	0.094	3.04	0.003
13 dpc_S	0.330	0.151	2.19	0.030
Day*Shedder infection group				
Effect of shedder group (1 vs 2) at				
5 dpc_S	0.893	0.331	2.70	0.008
7 dpc_S	0.021	0.194	0.11	0.912
9 dpc_S	− 0.219	0.130	− 1.69	0.093
13 dpc_S	− 0.311	0.100	− 3.10	0.002
Nasal swab				
Initial pig weight (log_10_ kg)	− 0.684	0.541	− 1.27	0.209
Shedder infection group (1 vs 2)	− 0.296	0.100	− 2.96	0.004
Shedder genotype (R− vs R+)	− 0.003	0.101	− 0.03	0.979
Day (relative to 7 dpc_S)^a^				
9 dpc_S	− 0.626	0.151	− 4.15	< 0.001
13 dpc_S	− 1.057	0.160	− 6.59	< 0.001

Estimate: Parameter estimate; se: standard error; t: t-value and 2 tailed p-value used in testing the null hypothesis that the coefficient/parameter is 0; R− and R+ : pigs with zero and one copy of the *WUR* resilience allele, respectively; dpc_S: Shedder pigs, days post contact from directly infected Inoculation pigs

^a^No nasal swabs were available at 5 dpc_S

The odds of having detectable virus in the nasal mucus at any sampling time-point did not differ significantly between the two R Shedder genotypes (p = 0.76) (Table 4). However, they tended to be higher for Shedder pigs from Shedder infection group 2 (p = 0.034) and on 7 dpc_S compared to 9 dpc_S and 13 dpc_S (Table 4).

**Table 4:** Estimated odds ratios and 95% confidence intervals (CI) for effects in the generalised linear mixed model for presence or absence of nasal shedding in Shedder pigs during the study

Effect	Fixed effects model				Odds ratio	
	Estimate	se	t	p-value	OR^**a**^	95% CI^**b**^
Initial pig weight (log_10_ kg)	− 4.521	2.851	− 1.59	0.115	0.011	< 0.001–3.07
Shedder infection group 1 vs 2	− 1.022	0.468	− 2.15	0.034	0.36	0.14–0.92
Shedder genotype R− vs R+	− 0.142	0.468	− 0.30	0.762	0.87	0.34–2.19
Day (relative to 7dpc_S)						
9 dpc_S	− 1.291	0.512	− 2.52	0.013	0.28	0.1–0.76
13 dpc_S	− 4.192	0.843	4.97	< 0.001	0.02	0.003–0.08

Estimate: Parameter estimate; se: standard error; t: t-value and 2 tailed p-value used in testing the null hypothesis that the coefficient/parameter is 0; R− and R+ : pigs with zero and one copy of the *WUR* resilience allele, respectively; dpc_S, for Shedder pigs, days post contact from directly infected inoculation pigs

^a^OR: odds ratio = exp(Estimate);

^b^95%CI for odds ratio = exp(Estimate ± 1.96*se)

In summary, no significant Shedder genotype effect was identified for any of the examined Shedder pig virus measures that would be indicative of a *WUR* genotype effect on Shedder pig infectivity.

### Indicators of shedder pig infectivity and susceptibility for Contact pigs

#### Contact pig infection status

Figure 4 shows the proportion of Contact pigs of each genotype that were infected by R+ and R− shedders at each sampling time (2 dpc_C, 7 dpc_C and 10 dpc_C), based on detectable virus in the serum of Contact pigs. Only 11 Contact pigs were infected at 2 dpc_C (contact genotype R+ n = 5/11; contact genotype R− n = 6/11). Contact pigs at 2 dpc_C were more likely to be infected by an R+ Shedder, although the number of infected Contact pigs was too small to achieve odd ratios that were statistically significantly different from 1 (R+ Contact pigs: n = 5/5 (100%), odds ratio (median Bayesian inference) 4.8 (0.36–1.75); R− Contact pigs: n = 4/6 (67%), odds ratio (median Bayesian inference) 0.21 (0.01–2.77)) (Fig. 4). In particular, all infected Contact pigs in Shedder Combination 2 were infected by an R+ Shedder, which originated from Shedder infection group 2 (Fig. 1).

There was no significant effect of the Contact pig R genotype (p = 0.61) on Contact pig infection status at 2 dpc_C (Table 5). These early infected Contact pigs tended to be heavier (p = 0.06) (Table 5). On 7 dpc_C and 10 dpc_C, respectively 96% (n = 92/96) and 99% (n = 95/96) of the Contact pigs were infected (Fig. 4). Across these two time-points there was no significant difference in the proportion of infected animals between the two R genotypes (Chi-square test: p = 0.88). Model results were similar for both time-points, so only the results for 10 dpc_C are presented (Table 5). The genotype of the transmitting shedder could be uniquely identified for all except three Contact pigs (see Additional file 7: Figure S9). Sixty percent of the R+ Contact pigs were infected by an R+ shedder pig, while equal proportions of R− Contact pigs were infected by an R+ and an R− Shedder pig (Fig. 4). However, statistically, the likelihood of infection from an R+ Shedder pig did not differ significantly between R+ and R− Contact pigs (Table 5; intercept p = 0.76). The likelihood of infection from an R+ Shedder pig increased if the cumulative R+ Shedder nasal levels in the room that they were housed in was higher (p = 0.08) (Table 5). Higher levels of nasal virus were detected in Shedder Infection group 2 on 7dpc_S (Fig. 3 and Table 3). R+ Shedder pigs from this Shedder Infection group were moved into Shedder Combination 2 (Fig. 1), which coincided with the fact that all infections on 2 dpc_C in Shedder Combination 2 were via R+ Shedder pigs. Hence, the apparent R+ Shedder effects on Contact pig infection status could be the result of the disproportionally large amount of virus that was shed by some R+ Shedder pigs in Combination 2 at the early stages post mixing.

**Table 5:** Odds ratios and 95% CI for based on the generalised linear mixed model for whether Contact pigs were infected at Day 2 dpc_C, and infected by an R+ shedder at Day 10 dpc_C

Effect	Fixed effects model				Odds ratio	
	Estimate	se	t	p-value	OR^**a**^	95% CI^**b**^
Infected at Day 2 dpc_C						
Intercept	– 6.069	2.68	– 2.27	0.033		
Initial Contact pig weight (kg)	0.538	0.281	1.91	0.060	1.71	0.98–3.00
Shedder combination 1 vs 2	0.152	0.665	0.23	0.820	1.16	0.21–4.39
Contact pig genotype R− vs R+	0.346	0.669	0.52	0.606	1.41	0.373–5.36
Shedder nasal shedding^c^	0.058	0.128	0.45	0.654	1.06	0.82–4.39
Infected by R+ shedder at Day 10 dpc_C^d^						
Intercept	0.480	1.20	0.40	0.694		
Initial contact pig weight (kg)	− 0.168	0.183	–0.92	0.363	0.846	0.59–1.22
Shedder Infection group 1 vs 2	− 0.302	0.437	–0.69	0.492	0.739	0.309–1.77
Contact pig genotype R− vs R+	− 0.634	0.444	–1.43	0.158	0.531	0.219–1.288
R+ Shedder nasal shedding^e^	0.108	0.061	1.76	0.083	1.114	0.986–1.26

Estimate: Parameter estimate; se: standard error; t: t-vaue and 2 tailed p-value used in testing the null hypothesis that the coefficient/parameter is 0; R− and R+ : pigs with zero and one copy of the *WUR* resilience allele, respectively; dpc_C, for Contact pigs, days post contact from directly infected Shedder pigs

^a^OR: odds ratio = exp(Estimate);

^b^95%CI for odds ratio = exp(Estimate ± 1.96*se)

^c^Total PRRSV-2 nasal shedding (sum log_10_TCID_50_) for all Shedder pigs in the room at 7 dpc_S

^d^On day 10 dpc_C 99% of the contact pigs were infected

^e^Total PRRSV-2 nasal shedding (sum log_10_TCID_50_) for R+ Shedder pigs in the room across all time points

#### Contact pig infection severity (AUC_C_ and log_10_TCID_50_ serum viral load)

Analysis of the AUC_C_ and log_10_TCID_50_ showed that there was no significant effect of the genotype of Contact pigs on their viral load (AUC_C_, p = 0.36; log_10_TCID_50_, p = 0.71), regardless of the R genotype of the Shedder pig that transmitted the infection. However, Contact pigs infected by a R+ Shedder pig had on average a higher AUC_C_ (p = 0.017) and log_10_TCID_50_ (p = 0.034) than Contact pigs infected by a R− Shedder pig (Table 6).

**Table 6:** Estimates from the linear mixed model of the effects on area under the curve (AUC_C_) and Log_10_TCID_50_ of Contact pigs from serum samples

Effect	Estimate	se	t	p-value
Area under the curve (AUC_C_)				
Initial pig weight (kg)	0.755	0.582	1.30	0.199
Shedder combination 1 vs 2	−0.661	1.32	−0.50	0.617
Infected by R+ Shedder^a^				
No vs Yes	−3.37	1.39	−2.42	0.017
Contact genotype				
R− vs R+	−1.21	1.30	−0.93	0.355
Shedder nasal shedding^b^	−0.083	0.500	−0.55	0.583
Log_10_TCID_50_ (day 7 and 10 dpc_C)				
Initial pig weight (kg)	0.045	0.054	0.83	0.409
Day (relative to 10 dpc_C) 7 dpc_C	−0.283	0.060	−4.71	< 0.001
Shedder combination 1 vs 2	0.014	0.115	0.130	0.900
Infected by R+ Shedder^a^				
No vs Yes	−0.260	0.121	−2.14	0.034
Contact genotype				
R− vs R+	−0.042	0.113	−0.37	0.712
Shedder nasal shedding^b^	−0.011	0.013	−0.850	0.399

Estimate: Parameter estimate; se: standard error; t: t-value and 2 tailed p-value used in testing the null hypothesis that the coefficient/parameter is 0; R– and R+ : pigs with zero and one copy of the *WUR* resilience allele, respectively; dpc_C, for Contact pigs, days post contact from directly infected Shedder pigs

^a^Both R+ and R− Shedder pigs genotypes were detected in three pigs. These pigs were removed from the analysis along with the single uninfected contact pig

^b^Total PRRSV-2 nasal shedding (sum log_10_TCID_50_) for Shedder pigs in the room across all time points

The cumulative Shedder nasal virus load in the room had no significant effect on the severity of infection of the Contact pigs. However, there was a significant effect of day (p = 0.004), with serum levels being significantly lower at 7 dpc_C than at 10 dpc_C (Table 6). Only 11 contact pigs were infected at 2 dpc_C and, thus, data from this sampling time were not included in the repeated measures model.

In summary, by combining results across the diverse Contact pig indicators, no statistically significant differences between R− and R+ Contact pigs were detected in either their probability or severity of infection that would indicate a *WUR* genotype effect on Contact pig susceptibility. However, there was some evidence that Shedder pigs from Shedder Infection group 2 were more infectious before 2 dpc_C, as all the contact pigs infected at 2 dpc_C in Shedder Combination 2 were infected by R+ shedders (from Shedder Infection Group 2). This may explain why infected Contact pigs experienced higher serum viral load (AUC_C_ and log_10_TCID_50_) if infected by an R+ Shedder pig, and the apparent Shedder genotype effect on Contact pig infection severity.

## Discussion

Breeding for disease resilience is most effective if it also simultaneously reduces pathogen transmission [14]. The aim of this study was to examine whether the previously identified *WUR* SNP associated with resilience of pigs to PRRSV infections [8] and to a poly-microbial natural pathogen challenge that included PRRSV [11] also confers differences in susceptibility and/or infectivity to pigs under natural horizontal PRRSV transmission. For this reason, a two-step trial was conducted, in which both Shedder and Contact pigs were infected through natural virus transmission by contact. Disentangling genotype or other fixed effects on host susceptibility and infectivity is complex and usually requires individual infection records for many contact groups [28, 29]. Furthermore, there is a risk that the effects of a specific genotype are confounded with effects of other genes if relatedness is not properly accounted for in the experimental design. A recently developed online statistical power analysis tool [30] suggests that at least eight contact groups comprising 12 shedder and 24 contact pigs would be required in order to detect a 10% or larger difference in susceptibility associated with the *WUR* SNP with 95% credibility, and at least 10 contact groups would be required for detecting the equivalent *WUR* SNP effect on infectivity. This online tool calculates the precision of the SNP effects under the assumption that genotype specific transmission routes are unknown and related individuals are distributed randomly across groups. Our study demonstrates that informative estimates of genotype effect for both host traits associated with disease transmission, i.e. susceptibility and infectivity, can be obtained from an even smaller-scale transmission experiments involving ~ 160 pigs and only four contact groups by balancing family relatedness and genotype of pigs at a putative resistance QTL in the different contact groups, and by barcoding the virus to trace pig genotype-specific transmission routes.

This study found no supportive evidence that genetic selection for the *WUR* PRRS resilience R+ genotype would also reduce PRRSV-2 transmission. In particular, we found no evidence that the *WUR* SNP confers differences in host susceptibility under natural PRRSV-2 transmission. All pigs used in our study became infected, except for one Contact pig (R− genotype). There was no apparent difference between the two host genotypes in overall serum virus load (Shedder and Contact pigs), as measured by AUC, or in log_10_TCID_50_ across sampling time points (Shedder and Contact pigs), or in time of infection (Contact pigs only) that would indicate a *WUR* genotype effect on Shedder pig infectivity or Contact pig susceptibility. Furthermore, no significant Shedder genotype effect was identified for nasal viral load of Shedder pigs that would be indicative of a *WUR* genotype effect on Shedder pig infectivity. However, inspection of the early infection status and severity of the Contact pigs indicated that more Contact pigs were infected by R+ Shedder pigs at the first sampling event and Contact pigs infected by an R+ Shedder pig had higher AUC_C_ and log_10_TCID_50_. This coincided with the fact that Shedder pigs from Shedder Infection Group 2 tended to have higher levels of nasal shedding at 7 dpc_S, at a time when the Shedder pigs were first introduced into the rooms with the Contact pigs (Table 3). Hence it is likely that the level and timing of nasal shedding by a small number of more infectious R+ shedder pigs in infection group 2 caused the observed differences in severity. A larger study with more replicates, in particular more Shedder Infection Groups, would be required to investigate the effects of shedding variability, and the presence of ‘super-shedders’ of a given genotype, on Contact pigs infection patterns.

Although this study provided conclusive results, there are several possible reasons that could have masked or inflated the *WUR* genotype effects on host susceptibility and infectivity. First, the PRRSV-2 (SD09-200) isolate that was used in this study has been circulating on North American farms and is known to cause mild infections, and infection doses in natural infections are expected to be lower than the inoculation doses in the PHGC challenge trials that led to identification of the *WUR* resilience SNP [15]. As a result, genetic differences in host response to counteract virus replication may be less pronounced in natural conditions. Indeed, sibling group variation in the AUC serum viral load of pigs in this study was relatively low, providing limited evidence for genetic variation in viral load of PRRSV-2 infected pigs [the random sibling group effect was not statistically significant based on log-likelihood ratio tests (see Additional file 4: Tables S5 and S6)]. This is in stark contrast to the compelling evidence for genetic differences in serum AUC that were obtained from the PHGC trials [8]. Second, the low virulence of the PRRSV-2 strain used in our study may dampen the effects of the *WUR* genotype on host susceptibility and infectivity. It has been shown that infection of susceptible pigs with highly pathogenic isolates results in higher viral concentrations in blood and tissues compared to pigs infected with mildly virulent isolates [2, 31] and that pathogenicity of the PRRSV strain enhances the effects of the *WUR* genotype on viral load [26]. Third, the sampling times may not have been ideal to reveal genotype effects on host transmission traits. The timing of sampling events was chosen to mimic previous PRRSV challenge studies, which highlighted different stages in the infection process, including the time of peak viremia (approximately 6 dpi) and the mid-to-late stage of infection (approximately 6–19 dpi) [25, 26]. However, these measures likely differ for different host and virus strains [26]. In our study, very few contact pigs were infected at 2 dpc_C but all except one were infected by 7 dpc_C. As a result, we did not fully capture differences in infection times of contact pigs to accurately determine to what extent these are affected by the *WUR* genotype of the contact or shedder pig. Future studies should increase the sampling frequency at the early stages of transmission to increase the chance to capture host genotype effects on susceptibility and infectivity.

Nasal mucus and serum samples were collected in this study. It is generally assumed that infectiousness is related to pathogen shedding rates but very few studies have set out to look directly at this relationship. Compared to serum samples, nasal swabs are a crude sampling method due to variation in the amount of starting material deposited on the swab and in collection depth, which are difficult to control. However, in an individual transmission study with the H1N1 pandemic swine flu strain, virus titre from nasal swabs was the most important predictor of transmission events, and transmission was more likely when nasal viral load was higher in shedder pigs [32]. The cumulative nasal viral load of Shedder pigs per room did improve the fit of all the models in our study but had no significant effect neither on the Contact pig probability of infection nor on their severity of infection (as measured by serum AUC_C_ and log_10_TCID_50_) (Tables 5 and 6). Furthermore, no difference in nasal virus load between the Shedder genotypes was observed in our study that could explain the observed Shedder genotype type effects on serum AUC_C_ and log_10_TCID_50_ of the Contact pigs. As a result, it appears that nasal viral load was not a reliable indicator to reveal potential differences in Shedder pig infectivity in this study.

In Contact pigs, the simultaneous presence of viruses from both R Shedder pig genotypes was more common in the nasal mucosa than in the serum, which suggests that the presence of virus in nasal swabs may be the result of contact between infected pigs. The presence of virus from different R genotypes in the nasal swab compared to the serum of the same pig indicates that contact does not always lead to transmission. As a result, nasal mucosa is not necessarily a reliable indicator of the transmission route. Although nasal viral load was found to be positively correlated with serum virus levels for both Shedder and Contact pigs, it was also more variable than serum viral load. In this study, although all (except one) pigs were viremic, only between 6 and 62.5% of the pigs appeared to be shedding the virus at each sampling event. In particular, nasal viral load was below detection level in 66% of pigs, although a few pigs shed at high levels. These high shedders may have contributed disproportionally to PRRSV-2 transmission in this study. As outlined above, the presence of such ‘super-shedders’ of a specific genotype could mask or inflate genotype effects, especially in small-scale experiments that involve relatively few infected shedders, as was the case in this study.

Future studies should focus on investigating the genetic architecture of susceptibility and infectivity of pigs for PRRSV or other pathogens as an important component for reducing disease transmission through genetic selection. In particular, in order to prevent undesirable side effects, such as accidental selection for tolerant super-spreaders [33], the role of the identified disease resistance, tolerance, or resilience SNPs or genes on disease transmission should be assessed before these genes are integrated into selection schemes. In particular, the genomic region tagged by the *WUR* SNP includes several candidate immune genes, including five members of the guanylate binding protein (GBP) family, which is known to be involved in immune regulation and in modulating the inflammatory response [8, 17]. Among these, a splice mutation in the *GBP5* gene was identified as the putative causative mutation at the *WUR* SNP [18]. A previous study showed that the *WUR* SNP is in high linkage disequilibrium (r^2^ = 0.94) with the *GBP5* gene [11], which suggests that it is unlikely that *GBP5* had a major effect on PRRSV transmission in this study. However, PRRS resilience is likely controlled by many genes, and several other candidate genes have been identified to be associated with host response to PRRSV [34]. Similar transmission experiments as that presented here could be carried out to assess the role of these loci in PRRSV transmission, or to assess whether pigs with high or low estimated breeding values for PRRS resilience differ in susceptibility or infectivity.

This study focused on identifying potential effects of the previously identified *WUR* resilience SNP on susceptibility and infectivity of pigs. However, host infectiousness depends not only on host infectivity but also on the duration of the infectious period [14]. For example, a recent study that assessed vaccine effects on PRRSV transmission found little evidence of lower PRRSV transmission rates in vaccinated pigs [35]. Nevertheless, PRRSV transmission was reduced, since vaccinated pigs, on average, experienced a shorter infectious period. Whether the *WUR* SNP affects the infectious period of PRRSV infected pigs is not known. However, previous studies have shown that pigs with the beneficial *WUR* resilience allele tended to clear the virus faster from blood [26], which suggests that these pigs may have a shorter infectious period and thus that selection for PRRSV resilience may indeed reduce PRRSV transmission.

Finally, it should be noted that in pig production systems, other sources of variation may contribute to PRRSV transmission, such as individual variation in contact behaviour between infected and non-infected pigs, or infections with other viruses or bacteria. Common pig behaviours such as fighting, tail-biting, and ear-biting can also result in more effective transmission [2]. Furthermore, aerosols or contact with contaminated materials are also known to play a role in PRRSV transmission [36, 37]. While genetic selection can potentially contribute to reducing disease transmission in farm animals, disease surveillance, biosecurity, and vaccination remain important for effective disease control.

## Conclusions

This study introduced a novel experimental design to assess host genetic effects on disease transmission. The experiment provided no supportive evidence that the previously identified *WUR* resilience SNP reduces PRRSV transmission. Given the relatively small scale of the present study and the potential implications of the results to the pig industry, it is of paramount importance that the results of this study are validated before the effects of the *WUR* SNP or *GBP5* gene on PRRSV transmission are dismissed.

## Supplementary Information

**Additional file 1:** **Text S1.** Pilot study to validate non-functional effects of barcoding. **Table S1.** Assignment of the pigs for the pilot study to test the effect of the 4 challenge versions of PRRSV-2 SD09-200. Three barcoded (BC1, BC2, BC3) and the parental wild type (WT) version, no barcoding.** Table S2.** Experimental design of the barcoding virus using PRRSV-2 strain SD09-200. **Table** **S3. **qRT-PCR primers and probes design for SD09-200 barcode virus. **Table S4.** Sequence primers for SD09-200 barcode viruses.

**Additional file 2:** **Text S2.**. Results from Pilot Study for viruses selected for the main study. **Figure S1.** Body temperature of piglets challenged with PRRSV-2. **Figure S2.** Viremia of piglets challenged with PRRS virus by cell culture. **Figure S3.** Viral titres in nasal swabs. **Figure S4.** Viral load (log_10_TCID_50_) in lung tissues at day 29.

**Additional file 3:** **Figure S5.** Serum PRRSV-2 viral load of the Inoculation pigs after they were exposed to the Shedder pigs.

**Additional file 4: Text S3. **Variation between and within sibling groups. **Table S5. **Variation across sibling groups for shedder pigs. Variation across sibling groups for contact pigs. **Table S6. **Shedder pigs—Log_10_TCID_50_ results for all shedder pigs used in the transmission experiment.

**Additional file 5: Figure S6.** Shedder pigs—Log_10_TCID_50_ results for all shedder pigs used in the transmission experiment.

**Additional file 6:** **Figure S7.** Shedder pigs—Boxplots of the distribution (log_10_TCID_50_) for serum samples. **Figure S8.** Shedder pigs—Boxplots of the distribution (log_10_TCID_50_) for nasal mucus samples.

**Additional file 7:** **Figure S9.** Contact Pigs. Log_10_TCID_50_ results for the serum and nasal swabs of all contact pigs used in the transmission experiment (n=96) for days 7 and 10 days post contact from the shedder pigs (dpc_C).

**Additional file 8:** **Figure S10.** Contact Pigs. Log_10_TCID_50_ results for the serum and nasal swabs of all contact pigs used in the transmission experiment (n=96) for days 7 and 10 days post contact from the shedder pigs (dpc_C).

## Data Availability

The datasets generated and/or analysed during the current study are not publicly available but are available from the corresponding author on reasonable request.
