# Supplementary material for: The WUR0000125 PRRS resilience SNP had no apparent effect on pigs’ infectivity and susceptibility in a novel transmission trial
Source: Genet Sel Evol. 2023 Jul 24;55:51. doi: 10.1186/s12711-023-00824-z (PMC10364427; doi:10.1186/s12711-023-00824-z)

**Additional file 1 Text S1**

1. **Pilot Study**

This text and associated tables and figures represent a summary of a pilot study that was completed prior to the main transmission trial. The objective of this study was to validate te non-functional effects of barcoding. The report on the pilot study is unpublished. If interested, an original copy may be obtained from the lead author.

**1.1 Study design**

**1.1.1 Challenge strains**

Two viruses were chosen for the main study, the PRRSV-SD09-200 (Group A, wild type strain) and PRRSV-SD09-200-BC3 (Group B, barcoding mutation) from the 4 viruses originally tested (Additional file 1, Table S1). The viruses were provided from Dr. Ying Fang at University of Illinois at Urbana-Champaign. The wild-type (WT) virus was isolated from a field sample in 2009 from South Dakota. The virus stocks and virus in the serum of the challenged piglets were sequenced (see description below). For more details on the methodology behind the barcoding (see [38]). Experimental design of barcode viruses, qRT-PCR primers and probes and recommended sequencing primers are shown in Additional file 1; Tables S2, S3 and S4 respectively.

**1.1.2 Total RNA Synthesis**

Nasal swabs and serum samples were thawed on ice and vortexed prior to total RNA extraction. The swabs were vortexed with the swab inside the 1.5 mL tube that contained 0.5mL of MEM (ThermoFisher Scientific, Gibco, 11095080) and centrifuged at 4000 rpm for 10 minutes at 25°C, and the used for RNA preparation. Carrier RNA was thawed at the time of RNA synthesis prepared and included according to the manufacturer’s instructions (QIAamp® Viral RNA Mini Handbook, Qiagen).

Total RNA was prepared from 150 µL of serum or nasal supernatant using the QIAmp Viral RNA kit (Qiagen, Cat#52906) and protocol. The final elution was performed with 50 µL nuclease free water. Samples were quantified using the Nanodrop2000 spectrophotometer and stored at -80°C. Samples were also cleaned up following RNA isolation using the RNeasy MinElute Cleanup Kit (Qiagen, Cat# 74204).

**1.1.3 cDNA synthesis**

Samples were briefly vortexed before performing the Superscript II First strand cDNA Synthesis protocol (ThermoFisher Scientific, Invitrogen, 18064014) using 500 ng of RNA, 50 ng/µL of random hexamers (ThermoFisher Scientific , Invitrogen, N8080127 ), 1 µL of dNTP Mix (10 mM each) and 12 µL of RNase/DNase free water were mixed on ice in a 0.2 mL PCR tube. The total mix was incubated at 65°C in a preheated thermocycler for 5 minutes, and immediately placed on ice for 1 minute. The tubes were centrifuged briefly prior to adding 4 µL of 5X First Strand Buffer (ThermoFisher Scientific, 18064014), 2 µL of 0.1M DTT, and 1 µL of RNAseOUT (ThermoFisher Scientific, Invitrogen, 10777019) at 40 units/µL and gently mixed together. Samples were centrifuged, and incubated for 2 minutes at 25°C, followed by the addition of 1 µL (200 units) of SuperScript II RT. The mixture was mixed by gentle pipetting, and incubated for 10 minutes at 25°C, before 50-minute incubation at 42°C. The reaction was terminated by incubation for 15 minutes at 70°C, and chilled on ice. Samples were diluted 1:2 and aliquoted in 8-strip tubes for qPCR analysis prior to being stored at -20°C.

**1.1.4 qPCR Analysis of Shedder and Contact nasal swabs and serum samples**

NFQ-MGB quencher probes for BC3 and WT PPRSV were designed and purchased from ThermoFisher Scientific using their custom probe design software. Probes were supplied as a 40X assay with the primers included and were diluted with TE Buffer pH 8.0 to a final concentration of 20X. A 50X Probe Master mix consisted of 250 µL of TnT Master mix, 100 µL of Nuclease free water, and 50 µL the probe (either WT or BC3). Strips were defrosted on ice for amplification.

Standards were designed for each probe (WT, BC3) and purchased from IDT Laboratories. TCID_50_ standards consisting of TCID_50_ 5.75 4.75, 3.75, 2.75, and 1.75 were prepared by serial dilutions in nuclease-free water. A concentration of 0.005ng/µl of gBlock was prepared using nuclease-free water. The TCID50 concentration of the diluted gBlocks was around 7. An additional 1:10 dilution was performed in a final volume 250µl. The TCID50 concentration of this diluted gBlock was around 6. TCID_50_ 5.75 standard was prepared by combining 224.8µl of the previous dilution with 175.2µl of nuclease-free water. The TCID_50_ 5.75 standard had a target Cq value of approx. 20, and standard dilutions had Cq values approximately 4 cycles apart, and were run in triplicate and averaged to determine this.

The qPCR run consisted of 8 µL of Master mix, and 2 µL of sample (or standard), plated in triplicate. Plates were vortexed and centrifuged prior to the assay on QuantStudio 3 (ThermoFisher Scientific), using the StepOne Plus Software. For cycle set up, ROX was assigned as the passive reference, and the assay ran at 95°C for 2 mins x 1 cycle; 95°C for 15 sec, 60°C for 60 sec x 40 cycles.

**1.1.5 qPCR data Analysis**

The ThermoFisher Connect online tool was used to obtain Cq values for each sample. The mean value for each sample and standard was calculated for each probe type. A sample was considered to be positive if the Cq value was recorded in 2 out of the 3 replicates. If there was a discrepancy between the 2 Cq values available, the samples were re-run, and this was done when values varied more than ±2.00. If a sample only had 1 replicate with a Cq value, the sample was scored as negative, and omitted from further analysis. A standard curve for WT and BC3 standards was generated using the line of best-fit function. Using the scatter plot function, the standard values were plotted on the y-axis, and the mean Cq values on the x-axis.

The r^2^-value and the equation for the line of best-fit were then calculated. The equation of the line (*y=mx+b*) was used for analysis, and the r^2^-value was confirmed to be above 0.99. The viral titres for each sample were then calculated by replacing the mean Cq value with x in the equation.

**1.1.6 Animal trial**

Sixteen piglets (8 pairs of full-sibs from 4 unrelated sires mated to 7 dams) were assigned into 4 groups (n=4) (see Additional file 1 Table S1). Age of piglets was about 4 weeks. Each group was housed in a separate animal room. On arrival, all animals were given one dose of Excede (Ceftiofur Crystalline Free Acid Zoetis®) as per label instructions. Animals were provided five days to acclimate before the start of the study. The study duration was 29 days.

On Day 0, Animals were challenged with PRRS viruses at a dose of 10^5^ TCID_50_ in a total volume of 4 mL (2 mL I.M. and 1 mL for each nostril). Serum samples and nasal swabs were collected for analysis of viral titres on days 0, 4, 7, 11, 14, 21 and 29 post challenge. Animals were observed daily for clinical presentations and clinical scores. Body temperature was measured daily for 7 days post-challenge. At necropsy, lung gross pathology was observed and lung lesion scores were assessed. Lung tissues were collected using a biopsy punch and used for viral load testing (29 dpc only).

**Additional file 1 Table S1**

**Assignment of the pigs for the pilot study to test the effect of the 4 challenge versions of PRRSV-2 SD09-200.Three barcoded (BC1, BC2, BC3) and the parental wild type (WT) version, no barcoding.**

| **PigID** | **Group** | **SowID** | **SireID** | **Challenge strain** | **Mutation present^a^** | **Used in the main study^b^** |
| --- | --- | --- | --- | --- | --- | --- |
| 1 | A | 1 | 1 | PRRSV-2 SD09-200-WT | No | Yes |
| 2 | A | 1 | 1 | PRRSV-2 SD09-200-WT | No | Yes |
| 3 | A | 2 | 2 | PRRSV-2 SD09-200-WT | No | Yes |
| 4 | A | 2 | 2 | PRRSV-2 SD09-200-WT | No | Yes |
| 5 | B | 6 | 3 | PRRSV-2 SD09-200-BC3 | Yes | Yes |
| 6 | B | 6 | 3 | PRRSV-2 SD09-200-BC3 | Yes | Yes |
| 7 | B | 7 | 1 | PRRSV-2 SD09-200-BC3 | Yes | Yes |
| 8 | B | 7 | 1 | PRRSV-2 SD09-200-BC3 | Yes | Yes |
| 9 | C | 3 | 3 | PRRSV-2 SD09-200-BC1 | No | No |
| 10 | C | 3 | 3 | PRRSV-2 SD09-200-BC1 | No | No |
| 11 | C | 4 | 4 | PRRSV-2 SD09-200-BC1 | No | No |
| 12 | C | 4 | 4 | PRRSV-2 SD09-200-BC1 | No | No |
| 13 | D | 5 | 3 | PRRSV-2 SD09-200-BC2 | No | No |
| 14 | D | 5 | 3 | PRRSV-2 SD09-200-BC2 | No | No |
| 15 | D | 6 | 3 | PRRSV-2 SD09-200-BC2 | No | No |
| 16 | D | 6 | 3 | PRRSV-2 SD09-200-BC2 | No | No |

^a^did the sequenced virus contain the expected barcode mutation

^b^only SD09-200-BC3 had the bar-coded mutations.SD09-200-BC1 and SD09-200-BC2 viruses did not have the expected bar-code mutations but were identical to the wild type strain SD09-200-WT.

**Additional file 1 Table S2**

**Experimental design of the barcoding virus using PRRSV-2 strain SD09-200.**


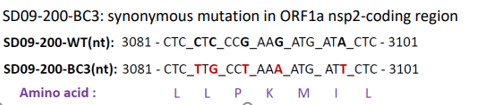


**Additional file 1 Table S3**

**qRT-PCR primers and probes design for SD09-200 barcode virus**


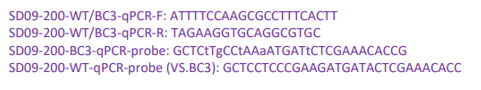


**Additional file 1 Table S4**

**Sequence primers for SD09-200 barcode viruses**


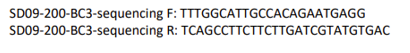

Supplement: Supplementary file 1 — Additional file 1: Text S1. Pilot study to validate non-functional effects of barcoding. Table S1. Assignment of the pigs for the pilot study to test the effect of the 4 challenge versions of PRRSV-2 SD09-200. Three barcoded (BC1, BC2, BC3) and the parental wild type (WT) version, no barcoding. Table S2. Experimental design of the barcoding virus using PRRSV-2 strain SD09-200. Table S3. qRT-PCR primers and probes design for SD09-200 barcode virus. Table S4. Sequence primers for SD09-200 barcode viruses. [file 12711_2023_824_MOESM1_ESM.docx]
