# Supplementary material for: The WUR0000125 PRRS resilience SNP had no apparent effect on pigs’ infectivity and susceptibility in a novel transmission trial
Source: Genet Sel Evol. 2023 Jul 24;55:51. doi: 10.1186/s12711-023-00824-z (PMC10364427; doi:10.1186/s12711-023-00824-z)

**Additional file 2 Text S2**

**2.0** **Results from Pilot Study for viruses selected for the main study**

**2.1 Clinical signs and body temperature**

No clinical signs were observed in any of the infected pigs. Lung lesion and clinical scores were 0 (normal) for all animals, every day. An increase in body temperature was seen in both groups one day post challenge. There were no significant differences in body temperature on any day between group A and Group B (see Additional file 3 Figure S1) (General linear model: day, p=0.372; group, p=0.395).

**2.2 Virus titres**

**2.2.1 Serum samples**

Viremia was seen in all animals from Group A and B on Day 4, 7, 11 and 14. There was no significant differences in serum viral load between the groups (Mann Whitney for each day: p>0.05; Additional file 3 Figure S2).

**2.2.2 Nasal swabs**

Viral titres from nasal samples (Additional file 3 Figure S3) were one to two orders of magnitude lower than those from serum (Additional file 3 Figure S2), and were below detection level for at least one pig in each Group on all days. There was no significant difference in viral titre between the two groups on day 4 and 7 (Mann Whitney: p>0.05).

**2.2.3 Lung tissue**

Virus titres in lung tissues were examined by cell culture (Additional file 3 Figure S4). Samples from 1 or 2 animals per group did not show viral load which could be due to the location of the tissues collected or could indicate there was no virus in the lung. There was no significant difference in the viral titre in the lung tissue among groups (Mann Whitney W=13.0, p=0.183)

**Additional file 2 Figure S1**

**Body temperature of piglets challenged with PRRSV-2**


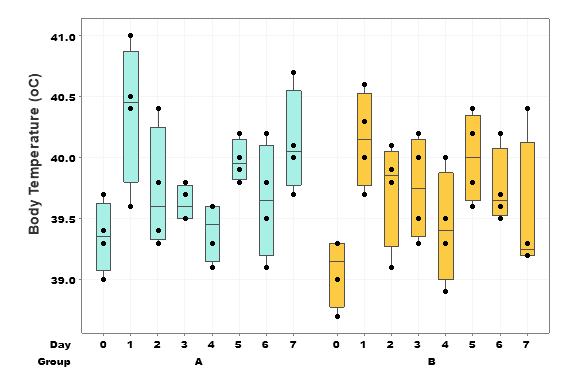
Group A, blue: SD09-200-WT; Group B, yellow: SD09-200-BC3 (with barcode mutations).

**Additional file 2 Figure S2**

**Viremia of piglets challenged with PRRS virus by cell culture**

Challenge day is Day 0 and all serum samples were PRRSV-2 negative. Group A, blue: SD09-200-WT; Group B, yellow: SD09-200-BC3 (with barcode mutations). Each dot represents one animal. Horizontal bars are median of the log_10_TCID_50_ values.


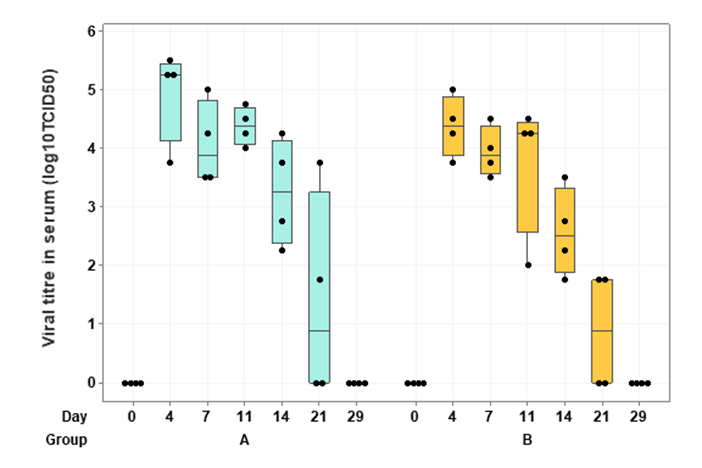


**Additional file 2 Figure S3**

**Viral titres in nasal swabs**

Group A, blue: SD09-200-WT; Group B: SD09-200-BC1 (wild type); Group B, yellow: SD09-200-BC3 (with barcode mutations). Each dot represents one animal. Horizontal bars are median of the log_10_TCID_50_ values.

**
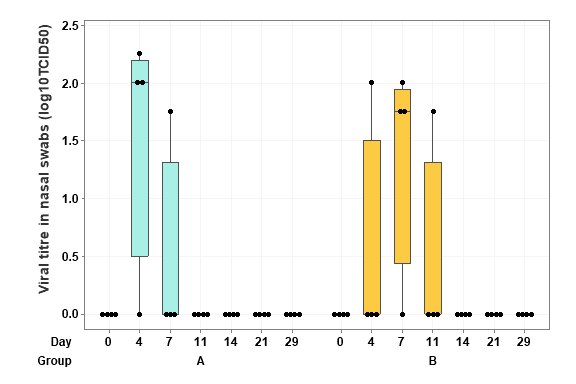
**

**Additional file 2 Figure S4**

**Viral load (log_10_TCID_50_) in lung tissues at day 29**

Group A, blue: SD09-200-WT; Group B, yellow: SD09-200-BC3 (with barcode mutations). Each dot represents one animal. Horizontal bars are median of the log_10_TCID_50_ values.


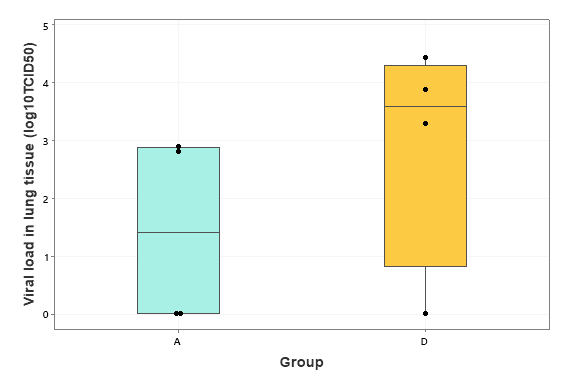

Supplement: Supplementary file 2 — Additional file 2: Text S2.. Results from Pilot Study for viruses selected for the main study. Figure S1. Body temperature of piglets challenged with PRRSV-2. Figure S2. Viremia of piglets challenged with PRRS virus by cell culture. Figure S3. Viral titres in nasal swabs. Figure S4. Viral load (log10TCID50) in lung tissues at day 29. [file 12711_2023_824_MOESM2_ESM.docx]
