# Supplementary material for: The WUR0000125 PRRS resilience SNP had no apparent effect on pigs’ infectivity and susceptibility in a novel transmission trial
Source: Genet Sel Evol. 2023 Jul 24;55:51. doi: 10.1186/s12711-023-00824-z (PMC10364427; doi:10.1186/s12711-023-00824-z)

**Additional file 3 Figure S5**

**Serum PRRSV-2 viral load of the Inoculation pigs after they were exposed to the Shedder pigs.**

The mean log_10_TCID_50_ (SD) in Room B (6.382 (0.067)) was slightly higher than in Room A (6.346 (0.068)) but the difference was not statistically significant (General Linear Model: df=1,18; f=1.41; p=0.250)


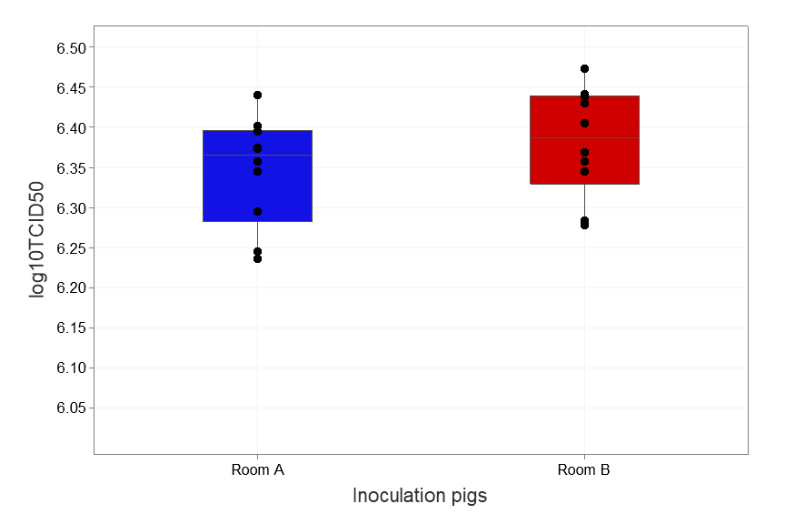

Supplement: Supplementary file 3 — Additional file 3: Figure S5. Serum PRRSV-2 viral load of the Inoculation pigs after they were exposed to the Shedder pigs. [file 12711_2023_824_MOESM3_ESM.docx]
