# Supplementary material for: The WUR0000125 PRRS resilience SNP had no apparent effect on pigs’ infectivity and susceptibility in a novel transmission trial
Source: Genet Sel Evol. 2023 Jul 24;55:51. doi: 10.1186/s12711-023-00824-z (PMC10364427; doi:10.1186/s12711-023-00824-z)
Supplement: Supplementary file 4 — Additional file 4: Text S3. Variation between and within sibling groups. Table S5. Variation across sibling groups for shedder pigs. Variation across sibling groups for contact pigs. Table S6. Shedder pigs—Log10TCID50 results for all shedder pigs used in the transmission experiment. [file 12711_2023_824_MOESM4_ESM.docx]

**Additional file 4 Text S3**

**3.0 Variation between and within sibling groups**

The genetics of the pigs used in this study was carefully managed. Sibling groups consisting mostly of 4 full siblings (or paternal half-sibs) with different R genotypes (2 R+ and 2 R-) were randomly assigned to different rooms to minimize the family effect. Sibling group was added to all models as it was part of the design of the study, accounting for the matching across replicates. To look at the variation between and within sibling groups, models for AUC were run with no random variables (No random effect) and then with sibling group as a random effect (Random, Sibling group). Improvement of model fit was examined using BIC and the log likelihood ratio test. Interclass correlation coefficient (ICC) was calculated to look at the proportion of the total variation explained by sibling group.

Variation across sibling groups was low, accounting for only 12% (shedder pigs) (Additional file 4, Table S5) and 14% (contact pigs) of the variation in serum AUC (Additional file 4, Table S6). The remaining variation (over 80%) is attributable to the individual pigs within the shedder pig and contact pig sibling groups. There was no systematic ranking of genotypes in the serum AUC. In particular R+ pigs did not have systematically lower AUC values than R- pigs (Fig. 2). The addition of sibling group improved the fit (lower BIC) of all models (Additional file 4, Table S5 and S6), but was not statistically significant according to the Log-Likelihood ratio test.

**Additional file 4 Table S5**

**Shedder pigs**

|  | No random effect | Random, Sibling group |
| --- | --- | --- |
| **A.Serum AUC** |  |  |
| *Fixed effects* |  |  |
| Intercept | 63.77(6.89) | 64.72 (7.26) |
| Log_10_ initial weight (kg) | -20.54 (9.67) | -21.94 (10.21) |
| Shedder group | 3.51 (2.00) | 3.52 (1.88) |
| Shedder genotype | 2.07 (2.02) | 2.11 (1.90) |
|  |  |  |
| *Sibling group variance* |  |  |
| between | - | 5.57 (7.14) |
| within | - | 42.42 (10.03) |
| ICC* | - | 0.12 |
|  |  |  |
| *Model fit* |  |  |
|  |  |  |
| -2logL | 322.0 | 321.2** |
| AIC | 332.0 | 333.2 |
| BIC | 341.4 | 336.1 |
| **B.Nasal swab AUC** |  |  |
| *Fixed effects* |  |  |
| Intercept | 52.00 | 52.21 (5.57) |
| Log_10_ initial weight (kg) | -7.70 | -8.00 (7.84) |
| Shedder group | -0.63 | -0.62 (1.48) |
| Shedder genotype | -0.10 | -0.92 (1.49) |
|  |  |  |
| *Sibling group variance* |  |  |
| between | - | 1.95 (3.80) |
| within | - | 26.18 (26.18) |
| ICC* | - | 0.07 |
|  |  |  |
| *Model fit* |  |  |
| -2logL | 296.4 | 296.1*** |
| AIC | 306.4 | 308.1 |
| BIC | 315.7 | 311.0 |

Linear model with Area Under the Curve (AUC) for serum (A) and nasal swabs (B) as the response variable, with (Random, sibling group) and without (No random effect) sibling group as a random effect in the model.*ICC (Interclass Correlation Coefficient) calculated as (between / between + within)

**log likelihood ratio test: p=0.2176

***log likelihood ratio test: p=0.3041

**Additional file 4 Table S6**

**Contact pigs**

|  | No random effect | Random, Sibling group |
| --- | --- | --- |
| *Fixed effects* |  |  |
| Intercept | 23.93 (6.49) | 23.63 (6.32) |
| Initial weight (kg) | 0.94 (0.57) | 0.98 (0.61) |
| Shedder combination | -1.00 (1.48) | -1.02 (1.38) |
| Shedder nasal shedding | -0.08 (0.28) | -0.07 (0.26) |
| Infected by R+ Shedder | -3.81 (1.47) | -3.79 (1.43) |
| Contact genotype | -1.47 (0.28) | -1.47 (1.32) |
|  |  |  |
| *Error variance: sibling group* |  |  |
| between | - | 6.22 (4.91) |
| within | - | 39.16 (6.60) |
| ICC* | - | 0.14 |
|  |  |  |
| *Model fit* |  |  |
| -2logL | 625.4 | 623.1** |
| AIC | 639.4 | 639.1 |
| BIC | 657.2 | 648.5 |

Linear model with serum AUC as the response variable, with (Random, sibling group) and without (No random effect) sibling group as a random effect in the model.

*ICC (Interclass Correlation Coefficient) calculated as (between / between + within)

**log likelihood ratio test: p=0.1028
