# Supplementary material for: The WUR0000125 PRRS resilience SNP had no apparent effect on pigs’ infectivity and susceptibility in a novel transmission trial
Source: Genet Sel Evol. 2023 Jul 24;55:51. doi: 10.1186/s12711-023-00824-z (PMC10364427; doi:10.1186/s12711-023-00824-z)
Supplement: Supplementary file 5 — Additional file 5: Figure S6. Shedder pigs—Log10TCID50 results for all shedder pigs used in the transmission experiment. [file 12711_2023_824_MOESM5_ESM.docx]

**Additional file 5 Figure S6**

**Shedder Pigs - Log_10_TCID_50_ results for all shedder pigs used in the transmission experiment.**


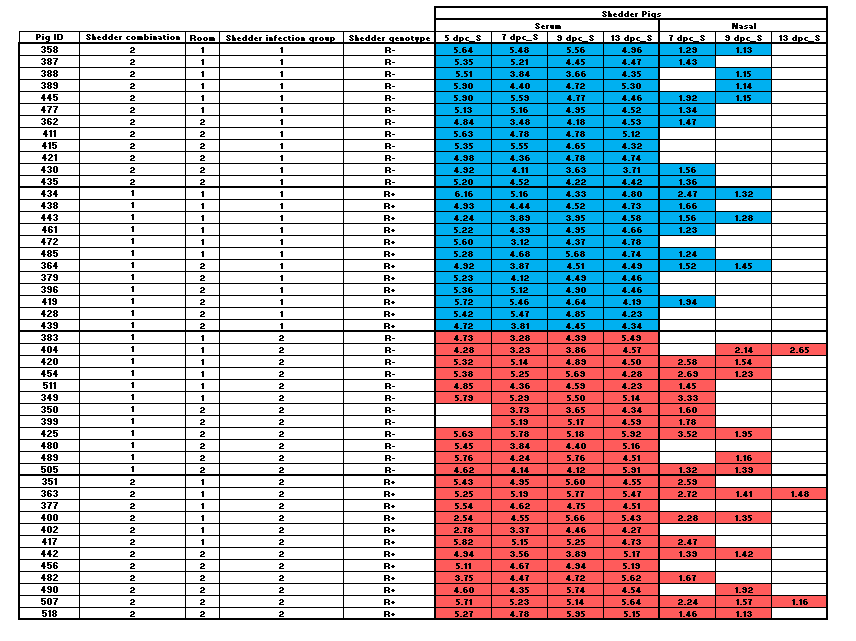
Coloured cells indicates that virus was detected in the sample. Values in the cells are the log_10_TCID_50_ value. The colour of the cells refers to the shedder infection group that the pig was infected with (serum) or shedding (nasal). Shedder infection group 1, blue; Shedder infection group 2, red. dps_S, days post contact from inoculator pig.
