# Supplementary material for: The WUR0000125 PRRS resilience SNP had no apparent effect on pigs’ infectivity and susceptibility in a novel transmission trial
Source: Genet Sel Evol. 2023 Jul 24;55:51. doi: 10.1186/s12711-023-00824-z (PMC10364427; doi:10.1186/s12711-023-00824-z)
Supplement: Supplementary file 6 — Additional file 6: Figure S7. Shedder pigs—Boxplots of the distribution (log10TCID50) for serum samples. Figure S8. Shedder pigs—Boxplots of the distribution (log10TCID50) for nasal mucus samples. [file 12711_2023_824_MOESM6_ESM.docx]

**Additional file 6 Figures S7**

**Shedder pigs - Boxplots of the distribution (log_10_TCID_50_) for serum samples**.


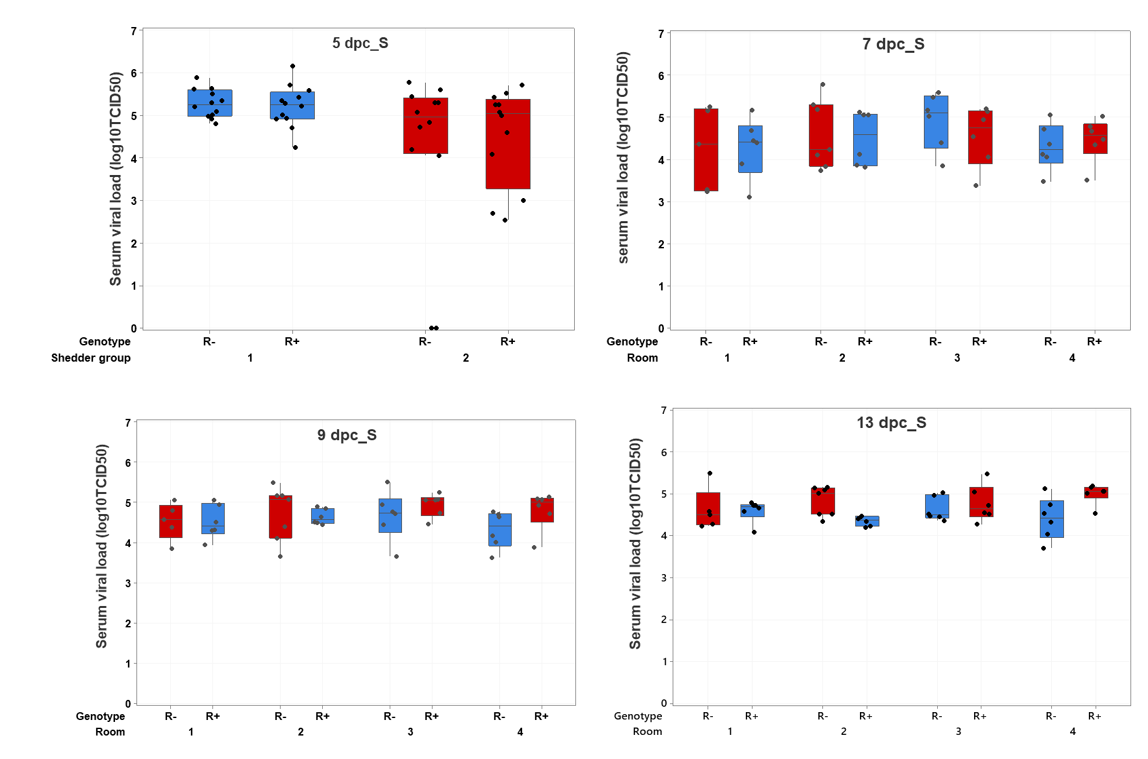
Blue, Shedder infection group 1; red, Shedder infection group 2. Day 5 dpc_S the Shedder pigs were still in their Shedder Infection Groups (see Fig. 1). Shedder pigs were moved on day 6 dpc_S. The day 7 dpc_S sample was taken after the Shedder pigs had been in contact with the Contact pigs for 1 day.

**Additional file 6 Figure S8**

**Shedder pigs - Boxplots of the distribution (log_10_TCID_50_) for nasal mucus samples.**


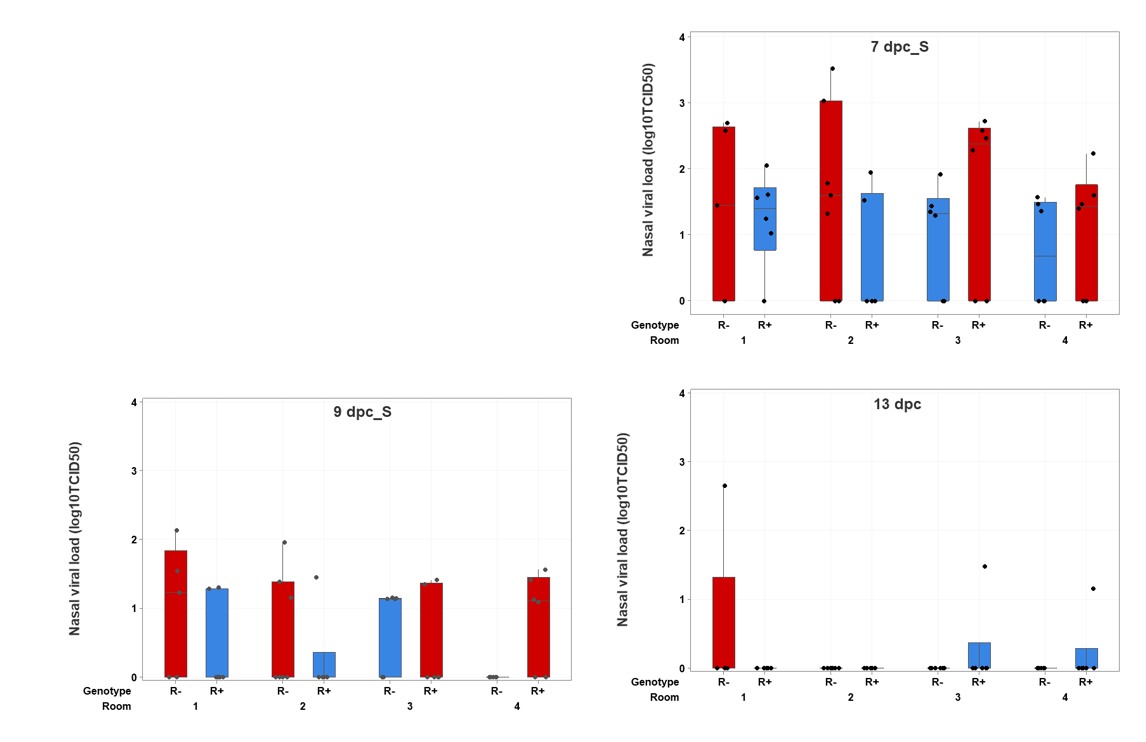
Blue, Shedder infection group 1; red, Shedder infection group 2. Day 5 dpc_S the Shedder pigs were still in their Shedder Infection Groups (see Fig. 1). Shedder pigs were moved on day 6 dpc_S. The day 7 dpc_S sample was taken after the Shedder pigs had been in contact with the Contact pigs for 1 day.
