# Supplementary material for: The WUR0000125 PRRS resilience SNP had no apparent effect on pigs’ infectivity and susceptibility in a novel transmission trial
Source: Genet Sel Evol. 2023 Jul 24;55:51. doi: 10.1186/s12711-023-00824-z (PMC10364427; doi:10.1186/s12711-023-00824-z)
Supplement: Supplementary file 7 — Additional file 7: Figure S9. Contact Pigs. Log10TCID50 results for the serum and nasal swabs of all contact pigs used in the transmission experiment (n=96) for days 7 and 10 days post contact from the shedder pigs (dpc_C). [file 12711_2023_824_MOESM7_ESM.docx]

**Additional file 7 Figures S9**

**Contact Pigs. Log_10_TCID_50_ results for the serum of all contact pigs used in the transmission experiment (n=96) for days 2, 7 and 10 days post contact from the shedder pigs (dpc_C).**


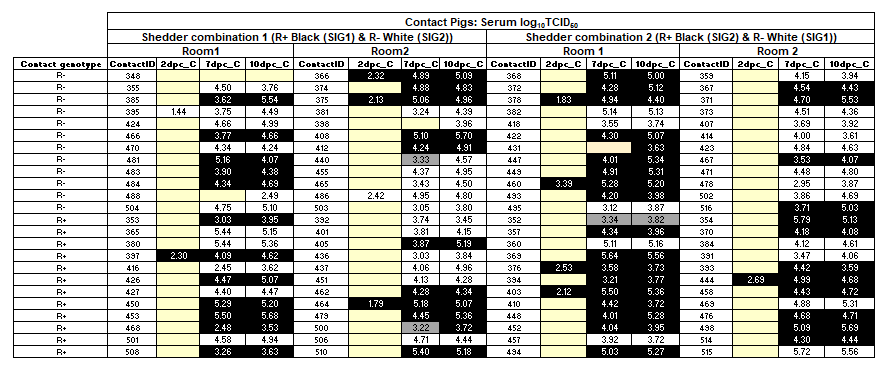
Yellow cells represent no virus detected (ND)). White cells are pigs that were infected by an R- shedder. Black cells are pigs that were infected by a R+ shedder. Grey cells are pigs that were infected by a R+ and a R- shedder. Values in the cells are the log_10_TCID_50_ values. SIG1, Shedder infection group 1; SIG2, Shedder infection group 2.
